# Detecting Solenoid Valve Deterioration in In-Use Electronic Diesel Fuel Injection Control Systems

**DOI:** 10.3390/s100807157

**Published:** 2010-07-29

**Authors:** Hsun-Heng Tsai, Chyuan-Yow Tseng

**Affiliations:** 1 Department of Biomechtronics Engineering, National Pingtung University of Science & Technology, Pingtung, 91207 Taiwan; 2 Department of Vehicle Engineering, National Pingtung University of Science & Technology, Pingtung, 91207 Taiwan; E-Mail: chyuan@mail.npust.edu.tw

**Keywords:** solenoid valve, diesel engine, fault detection, LVDT sensor

## Abstract

The diesel engine is the main power source for most agricultural vehicles. The control of diesel engine emissions is an important global issue. Fuel injection control systems directly affect fuel efficiency and emissions of diesel engines. Deterioration faults, such as rack deformation, solenoid valve failure, and rack-travel sensor malfunction, are possibly in the fuel injection module of electronic diesel control (EDC) systems. Among these faults, solenoid valve failure is most likely to occur for in-use diesel engines. According to the previous studies, this failure is a result of the wear of the plunger and sleeve, based on a long period of usage, lubricant degradation, or engine overheating. Due to the difficulty in identifying solenoid valve deterioration, this study focuses on developing a sensor identification algorithm that can clearly classify the usability of the solenoid valve, without disassembling the fuel pump of an EDC system for in-use agricultural vehicles. A diagnostic algorithm is proposed, including a feedback controller, a parameter identifier, a linear variable differential transformer (LVDT) sensor, and a neural network classifier. Experimental results show that the proposed algorithm can accurately identify the usability of solenoid valves.

## 1. Introduction

The control of engine emissions is an important global issue for on- and off-road vehicles. The fuel injection control system dramatically affects the fuel efficiency and the emissions of diesel engines in agricultural vehicles [1,2]. Recently, advancements in electronics and measurement technology have led to a substantial improvement of the fuel injection control, both in hardware configuration and control methodology. A typical example is the BOSCH electronically controlled P-EDC in-line fuel-injection pump. In this system, a linear solenoid valve, as opposed to a conventional mechanical governor, is used to actuate the control rack of the fuel pump to regulate the amount of injected fuel. A rack-travel sensor measures the rack position, which is corresponding to the amount of injected fuel. The electronic control unit (ECU) regulates the rack position to supply the desired amount of fuel. Because the amount of injected fuel has a substantial influence upon the engine’s performance, deterioration status of these main components dominates the emission level of in-use vehicles.

Rack deformation, solenoid valve deterioration, and rack-travel sensor malfunction are the possible deterioration faults in an EDC system. Among these faults, solenoid valve deterioration is most likely to occur and is one of the reasons causing a diesel engine to have a hunting phenomenon with high smoke emission levels and unstable idle speed. However, solenoid valve faults are difficult to diagnose due to lack of the mechanical or electrical damage signs on the solenoid valve [3–6]. In practice, only the plunger clearance and coil resistance of the solenoid valve can be measured as a reference to diagnose the usability. As an example, the acceptable values of the coil resistance and plunger clearance are 0.6–0.9 Ω and 0.12 mm respectively, for a BOSCH EDC system model. Unfortunately, the plunger clearance is difficult to measure for *in situ* diagnosis because the solenoid is mounted inside the pump, and furthermore it is a destructive diagnosis method with high service cost. Thus, a practical diagnosis method to detect the solenoid valve deterioration status without disassembling the pump is required.

Component fault detection and diagnosis (FDD) for vehicles has been studied for two decades. Examples include the observer-based approaches [7–10] and the parameter-estimation approaches [11–14]. These methods have been proven capable of detecting certain types of system faults. However, most of the previous works focused on diagnosing electrical faults in sensors or actuators. Works on mechanical fault diagnosis of actuators are very limited, especially for an EDC system.

Since an excessive plunger clearance of an EDC system’s solenoid indicates plunger or sleeve wear, it is suspected that solenoid valve deterioration may be caused by frictional forces. In [15], it was shown that solenoid valve failure is mainly due to the wear of the plunger and sleeve resulting from a long period of usage, lubricant degradation, or engine overheating. Such deterioration leads to a large amount of Coulomb frictional force in the solenoid valve and consequently results in its failure. Based on those results, a nondestructive technique that can clearly identify the usability of solenoid valve is developed in the current paper, where an investigation of the relationship between deterioration conditions and the malfunctions of the EDC system is reported. Some system parameters are identified to characterize the deterioration status. In addition, a neural network based classifier is applied to diagnose the status of the solenoid valve deterioration. The resulting methodology is intended to support both on-board and service applications.

This paper is organized as follows. The equation of motion of the system is analyzed in Section 2. In Section 3, the parameter identification is developed. The following section shows the experimental results. In Section 5, the deterioration fault detection based on a neural network is presented. Section 6 concludes this paper.

## 2. System Modeling

In a fuel injection control system, the motions of the solenoid valve, the rack, and its loads such as the injection plungers in the fuel pump, are governed by the interaction between the electromagnetic force, the spring force, and other resistive forces. When these forces are balanced, the rack position reaches its equilibrium. The dynamic equation for this system can be represented as follows:

(1) $$m\ddot{x}+c\dot{x}+\mathrm{kx}+F_{f}\;(\dot{x}(t),F_{m})=F_{m}$$

where *x* is the rack position, *F_f_* (*ẋ*,*F_m_*) represents the friction force and other un-modeled forces, *k* is the spring constant, *c* is the damping coefficient, *m* is the mass of the moving parts, and *F_m_* is the driving force of the actuator. When the actuator is excited by a time dependent voltage, *u*, the current developed in the coil windings is governed by:

(2) $$L\frac{\mathrm{di}}{\mathrm{dt}}+iR_{c}=k_{A}u(t)$$

where *R_c_* is the coil resistance, *L* is the coil inductance, and *k_A_* is the gain of power amplifier. The driving force *F_m_* in Equation (1) is a nonlinear function of the coil current and air gap. By linearizing *F_m_* around the operation point of the system, it yields:

(3) $$F_{m}=-k_{x}\;x+k_{i}\;i$$

In general, the most significant friction components in a servo-mechanical system are the static friction, the Coulomb friction, and the viscous friction. Thus, the friction force *F_f_* in Equation (1) can be formulated as:

(4) $$F_{f}\;(\dot{x},F_{m}\;)=F_{0}(\dot{x},F_{m})+F_{c}\;\text{sgn}(\dot{x})$$

where *F*_0_ and *F_c_* are the static and Coulomb friction forces, respectively. Here the viscous friction associated with the velocity is excluded because its effect is considered in the damping behavior of the system. In an EDC system, only the rack position *x*, the driving signal *u*, and the associated current *i* in equations (1–4) are available for diagnosis. Thus, an algorithm to identify the other necessary parameters for the diagnosis is required.

## 3. Strategies for Parameter Identification

When an actuator in a control system malfunctions, the dynamic characteristics of the system change accordingly. Therefore, this study proceeded by driving the the EDC system rack steadily using a sinusoidal reference input. To ensure the tracking is stable, a feedback controller is introduced. The parameters are then identified through a feedforward path in the system, which is a two-degree-of-freedom controller proposed by Sugie *et al.* [16]. Iwasaki *et al.* have successfully adopted this method in their position control mechanism [17]. In this section, the algorithm for parameter identification is derived and its characteristics are investigated.

**Figure 1.**
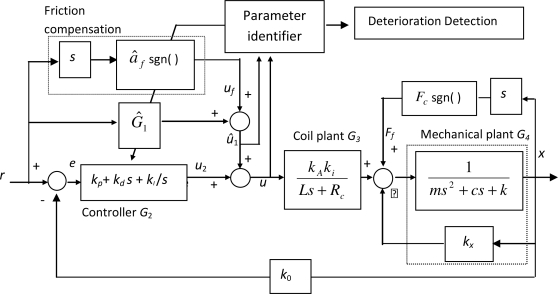
Block diagram of the proposed diagnostic system.

The block diagram of the proposed method is shown in Figure 1. *G*_4_ denotes the dynamics of the plant corresponding to the models in equations (1) and (3); *G*_3_ is the dynamic characteristics of the current flowing through the coil of the actuator in Equation (2), *G*_2_ is the feedback controller, *G*_1_ is the feedforward model, *r* is the position reference, *x* is the position output of the control plant, *u*_1_ is the output of *G*_1_, and *u*_2_ is the output of the feedback controller. In addition to *G*_1_, the friction compensation, *F*_comp_, is also considered in the feedforward loop. From Figure 1, the connections between *u*_2_, the friction force *F_f_* and the reference input *r* can be derived as follows [15]:

(5) $$u_{2}=\frac{G_{2}-k_{0}\;(G_{1}+F_{\text{comp}})G_{2}G_{3}G_{4}}{1+k_{0}G_{2}G_{3}G_{4}}r+\frac{k_{0}G_{2}G_{4}}{1+k_{0}G_{2}G_{3}G_{4}}F_{f}.$$

If *F*_comp_ and the parameters in *G*_1_ are identified properly such that the characteristics of the plant and the friction can be obtained, *i.e.*, if the following conditions:

(6) $$G_{1}={\left(k_{0}G_{3}G_{4}\right)}^{-1}$$

and:

(7) $$u_{f}=F_{\text{comp}}r=\frac{1}{G_{3}}F_{f}$$

are satisfied, the pump rack will track the desired trajectory, which is determined by the zero state errors of reference positions. Then, the feedback control effort *u*_2_ will become zero, and consequently the condition *u* = *u*_1_ will be achieved. Based on this idea, a parameter identification algorithm is proposed as follows: The output of the friction compensation in Equation (7) can be approximated by:

(8) $$\frac{1}{G_{3}}F_{f}\approx \frac{R_{c}}{k_{A}k_{i}}F_{c}\;\text{sgn}(\dot{r})=a_{f}\;\text{sgn}(\dot{r})$$

where $a_{f}=\frac{R_{c}}{k_{A}k_{i}}F_{f}$. Furthermore, *G*_1_ can be expanded as:

(9) $$G_{1}=a_{3}s^{3}+a_{2}s^{2}+a_{1}s+a_{0}$$

where *s* is the Laplace variable. Thus, the total feedforward compensation becomes:

(10) $$u_{1}=G_{1}r\;+\;a_{f}\;\text{sgn}(\dot{r})$$

Using equations (9) and (10), the total feedforward compensation can be estimated as:

(11) $${\hat{u}}_{1}={\hat{a}}_{3}\dddot{r}+{\hat{a}}_{2}\ddot{r}+{\hat{a}}_{1}\dot{r}+{\hat{a}}_{0}+{\hat{a}}_{f}\;\text{sgn}(\dot{r})$$

where *â_f_* and *â*_0–3_ are the parameters to be identified. From Figure 1, the values of *â_i_*’s are related to the physical parameters and can be represented as follows:

(12) $$\begin{array}{c} a_{3}=\frac{\mathrm{Lm}}{k_{A}k_{i}k_{o}},\;a_{2}=\frac{mR_{s}+\mathrm{Lc}}{k_{A}k_{i}k_{o}},\;a_{1}=\frac{L(k-k_{x})+R_{s}c}{k_{A}k_{i}k_{o}}\\ a_{0}=\frac{R_{s}(k-k_{x})}{k_{A}k_{i}k_{o}},\;a_{f}=\frac{R_{c}}{k_{A}k_{i}}F_{f} \end{array}$$

If the parameters are all identified correctly, *i.e.*, *â*’s = *a*’s, *u*_1_ = *u* will be achieved. Therefore, we make *u*_1_ equal to *u* to estimate the parameters in Model (11). The parameter identification error can be defined as:

(13) $$e_{2}=u-u_{1}=W\hat{a}-\mathrm{Wa}=W\tilde{a}$$

where $W={\left[\begin{array}{lllll} \dddot{r} & \ddot{r} & \dot{r} & r & \text{sgn}(\dot{r}) \end{array}\right]}^{T}$  and $\hat{a}={\left[\begin{array}{lllll} {\hat{a}}_{3} & {\hat{a}}_{2} & {\hat{a}}_{1} & {\hat{a}}_{0} & {\hat{a}}_{f} \end{array}\right]}^{T}$. With these notations, the gradient operator can be applied to obtain the following on-line parameters estimator:

(14) $$\dot{\hat{a}}(t)=-\mathrm{PW}(t)e_{2}\;(t)$$

where the scalar gain matrix *P* is a positive-definite matrix called the estimator gain. This on-line algorithm updates the estimation, *â*. By starting with an initial estimation *â*(0) and its corresponding *e*_2_(0), we can sequentially update *â* iteratively. Note that only the reference data are included in *W*. Thus, the identification is insensitive to disturbances.

## 4. Experiments

**Figure 2.**
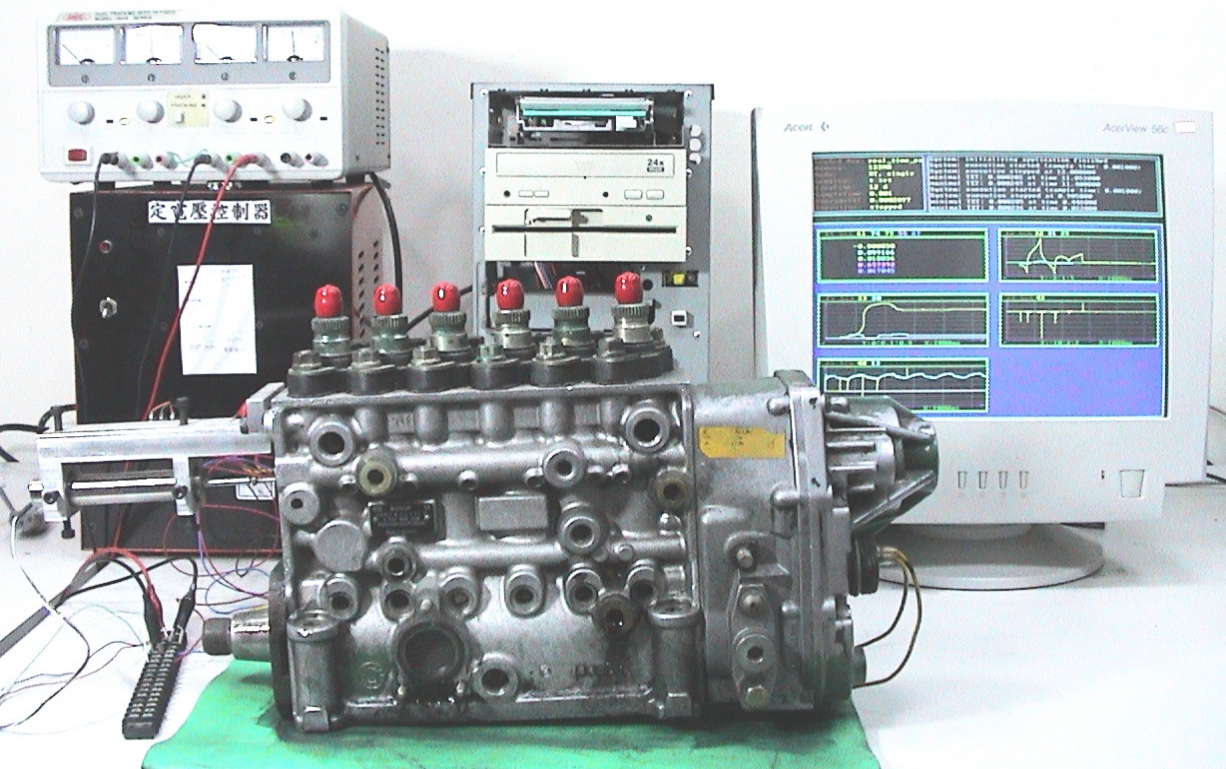
Experimental apparatus where a BOSCH P-EDC fuel pump is shown.

To study how the wear condition relates to the malfunction of EDC systems, several experiments were performed on a BOSCH P-EDC fuel pump as shown in Figure 2. Seventeen different solenoid valves collected from several diesel fuel pump service shops were used. Among these solenoid valves, four were brand new and the other thirteen presented different wear conditions. The experimental apparatus for this study, as shown in Figure 2, included a power amplifier, a controller and an EDC fuel pump equipped with a LVDT type position sensor. The controller was a personal computer installed with Matlab XPC real time control software. It consisted of a feedback controller, a feedforward parameter identifier, and a digital filter with a bandwidth of 20 Hz. A 0.5 Hz sinusoidal signal was used as the desired motion of the middle stroke of the solenoid.

The algorithm shown in Figure 1 was implemented as follows: the conventional PID controller is adopted as the feedback controller *G*_2_. Initially, *G*_1_ was set to zero and the gains in *G*_2_ were adjusted such that a stable rack motion can be achieved. Then the parameters in *G*_1_ were identified using equations (13) and (14). The tests were performed on the pump for solenoids with different degrees of wear using the same controller gains in *G*_2_. Before each test, the coil resistance and the clearance between the plunger and the sleeve of the solenoid were measured. According to the manufacturer’s specifications, the acceptable values of the coil resistance and plunger clearance were 0.6–0.9 Ω and 0.12 mm, respectively. In the experiments, the resistances of all the solenoid valves were acceptable, but the measured plunger clearances, depending on their period of usage, showed large variations. In the following paragraphs, three critical cases are presented for demonstration.

**Figure 3.**
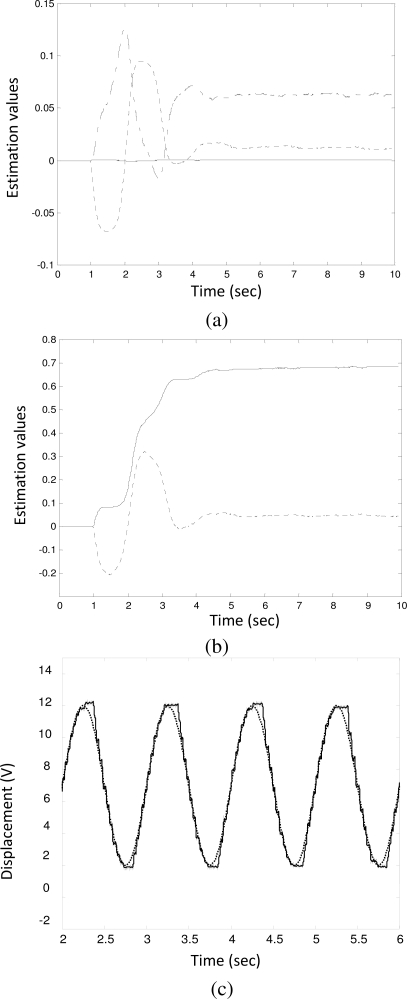
Parameter identification for valve V_1_. **(a)** *â*_3_: solid line, *â*_2_: dot-solid line, *â*_1_: dot line; **(b)** *â*_0_: solid line, *â_f_*: dot line; **(c)** the tracking signal (solid line) and the reference input (dot line) during identification.

Figure 3 shows the case for a brand new solenoid valve (denoted by V_1_). The plunger clearance and the coil resistance were measured as 0.09 mm and 0.7 Ω, respectively. The estimated value rapidly converged to its final value as follows:

$${\hat{a}}_{v1}={\left[0.0001,\;0.0630,\;0.0118,\;0.6819,\;0.0469\right]}^{T}$$

Although the system was equipped with a brand new solenoid valve, the overall system still experienced a small extent of friction (*â_f_* = 0.0469) caused by other mechanical components. Figure 3(c) shows that the rack tracks the reference input satisfactorily. However, as shown in Figure 3(c), due to the Coulomb friction, the rack trajectory (solid line) deviated slightly from the reference input (dot line) at the transition points. Note that *â_f_* is an equivalent value but not the true value of the frictional force. From Equation (12), the actual friction force *F_f_* is determined by:

$$F_{f}=\frac{k_{A}k_{i}}{R_{c}}{\hat{a}}_{f}$$

**Figure 4.**
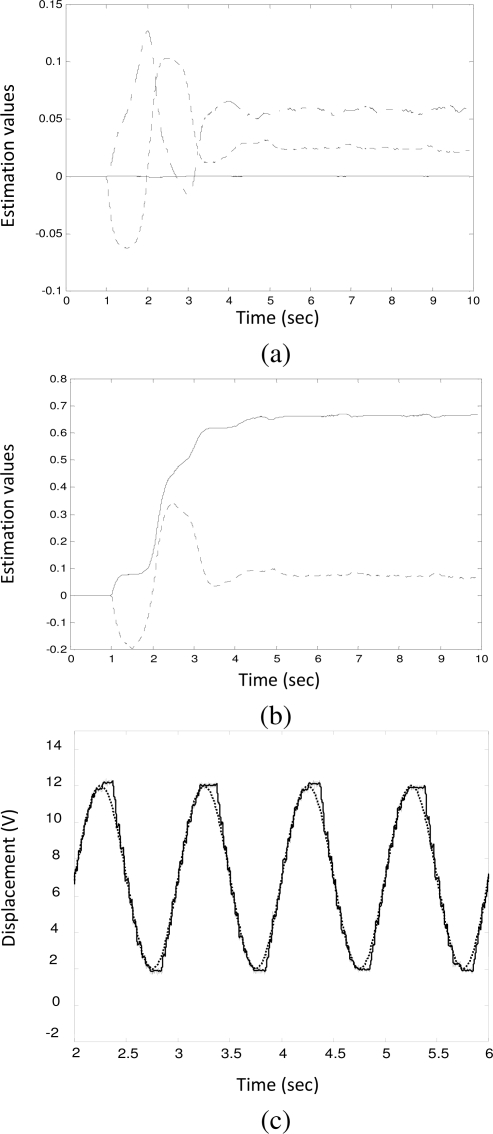
Parameter identification for valve V_2_. **(a)** *â*_3_: solid line, *â*_2_: dot-solid line, *â*_1_: dot line; **(b)** *â*_0_: solid line, *â_f_*: dot line; **(c)** the tracking signal (solid line) and the reference input (dot line) during identification.

Figure 4 shows the identification results for a pump equipped with a worn solenoid valve (denoted by V_2_). It was disassembled from a vehicle which produced a certain amount of smoke emissions, but its idle speed could still be stabilized. The coil resistance and the plunger clearance were measured as 0.7 Ω and 0.25 mm, respectively. The parameters were obtained as:

$${\hat{a}}_{v2}={\left[0.0001,\;0.0583,\;0.0239,\;0.6649,\;0.0715\right]}^{T}$$

Due to the wear, the plunger clearance increased to 0.25 mm and the identified friction coefficient (*â_f_*) increased to 0.0715. The effect of the increased friction can be clearly observed from Figure 4(c) in which the rack trajectory (solid line) and the reference input (dotted line) were shown. Due to friction force, the rack motion exhibited a chattering phenomenon.

**Figure 5.**
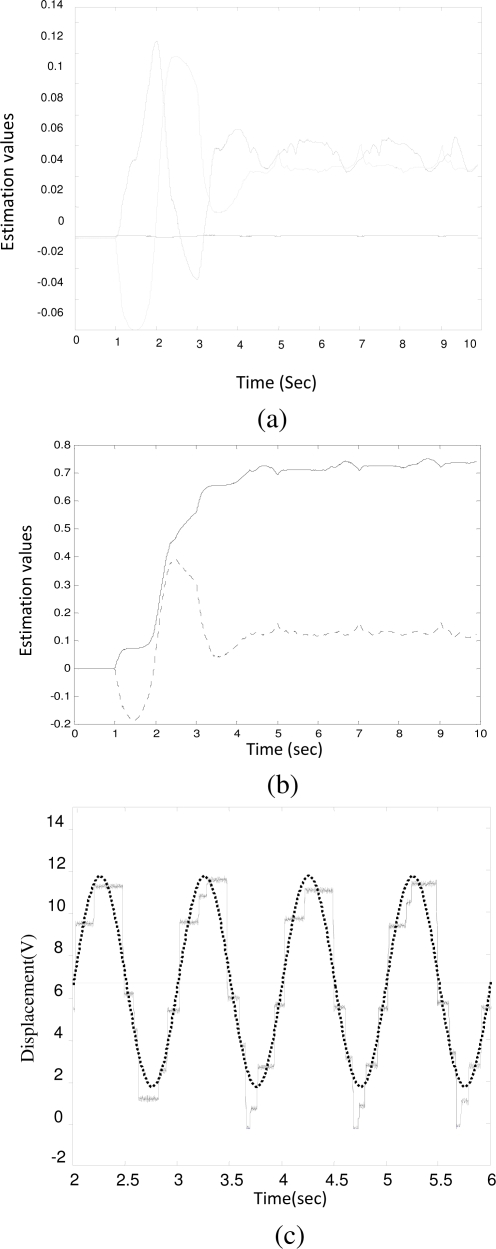
Parameter identification for valve V_3_. **(a)** *â*_3_: solid line, *â*_2_: dot-solid line, *â*_1_: dot line; **(b)** *â*_0_: solid line, *â_f_*: dot line; **(c)** the tracking signal (solid line) and the reference input (dot line) during identification.

Figure 5 indicates the identification results for another worse scenario. Here, a faulty solenoid valve (V_3_) with a service life over 97,000 km was used. The coil resistance and the plunger clearance were measured as 0.7 Ω and 0.2 mm, respectively. The smoke emission level from the diesel engine was too high to pass the EPA standard in Taiwan and, furthermore, there is a hunting phenomenon coming along with it. When this solenoid valve was fitted on the test pump, the identified parameters were obtained as:

$${\hat{a}}_{v3}={\left[0.0001,\;0.0546,\;0.0471,\;0.7321,\;0.1284\right]}^{T}$$

Although the plunger clearance (0.2 mm) was less than that of V_2_, it still produced a greater frictional force (*â_f_* = 0.7321). It is believed that the rough surface between the plunger and sleeve of the solenoid valve due to uneven wear attributed to the increase of friction. As shown in Figure 5(c), the chattering phenomenon was more dramatic than that of V_2_.

By comparing the identified parameters of the worn solenoid valves (V_2_ and V_3_) with that of the brand new one (V_1_), the percentage increases on *â*_0−3_ and *â_f_* were obtained and listed in Table 1. Here, only the two critical cases are shown for simplicity; the other cases had the same trend similar to these two cases. As seen in this table, in addition to *â_f_*, the wear of the solenoid valve also induced significant changes on *â*_1_. On the other hand, the changes on *â*_0_, *â*_2_, and *â*_3_ were not significant. This is because the wear of the solenoid valve increased the damping force of the valve plunger which results in an increase of *â*_1_. Therefore the fault of a pump rack control system mainly resulted from the wear of solenoid valve, and this kind of fault can be diagnosed by monitoring the values of *â_f_* and *â*_1_.

## 5. Deterioration Detection

As presented in Section 4, since the vector (*â_f_*, *â*_1_) characterizes the wear condition of the solenoid valve, the solenoid valve failure due to the wear can be diagnosed by monitoring the variation of this vector. Thus, the diagnostic work should focus on the value of (*â_f_*, *â*_1_) instead of the system parameters. This reduces the dimensionality of the input data for diagnosis and the computation time can be reduced significantly. Note that the changes in the physical parameters still cannot be detected, since the number of model parameters is less than that of the physical parameters. For diagnostic applications, however, it is not necessary to display the changes in the physical parameters. Only a malfunction signal to trigger an alarm is needed. In other words, a decision boundary to classify the faulty component is required. This leads to a two-dimensional two-class classification problem with only one decision boundary necessary [18].

**Figure 6.**
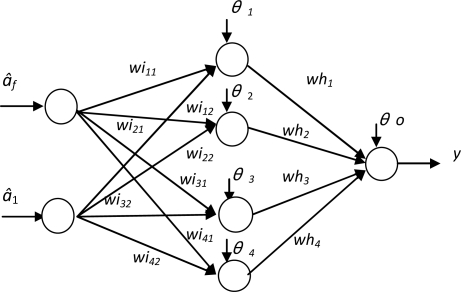
Configuration of the neural network.

**Figure 7.**
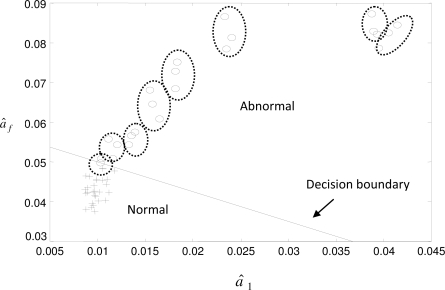
The decision boundary that partitions the input space (*â_f_*, *â*_1_) into regions corresponding to normal and abnormal components. The dashed circles that encircle the data points belong to the same solenoid. The solenoids classified as normal are marked with “+”, while the abnormal solenoids are “O”.

In this section, an artificial neural network (ANN) was used for the classification purpose. The network was a three-layer (including the input and output layers) feedforward network with nonlinear hidden and output units, as shown in Figure 6. In this network, the bias values *θ_i_*’s, weights *wh_i_*’s and *wi_ij_*’s were assigned using a generalized back propagation training algorithm. The training in this study was performed off-line utilizing a previously generated training data which consists of input values of (*â_f_*, *â*_1_) and their corresponding outputs (*y*) in terms of Boolean values (0/1) indicating whether the solenoid is normal or abnormal. Six solenoids were utilized to train the network. Among these solenoids, two were severely worn, whereas the others were brand new. Each of these solenoids was tested three times, and total of 18 input/output patterns were obtained. After training, the ANN forms a decision boundary that partitions the input space (*â_f_*, *â*_1_) into regions corresponding to normal and abnormal components, shown in Figure 7.

Another eighteen old solenoids were used to test the effectiveness of the classification. Of these old solenoids, ten were reusable and eight were faulty. In this figure, the solenoids classified as normal are marked as “+”, while the abnormal solenoids are marked as “O”. Since each solenoid was tested three times, the three data points belonging to the same solenoid were circled by a dash line. As seen in this figure, the ten reusable solenoids were classified into the normal region. For the eight faulty solenoids, seven were successfully classified into the abnormal region except the one close to the decision boundary.

Two data points of this solenoid were classified in the abnormal region while one was in the normal region. This indicates that its wear condition is not as severe as the others, but still should be classified as an abnormal component. As shown in Figure 7, the data points belonging to the same solenoid (in the same dashed circle) are evidently very close to each other. This fact reveals the reproducibility of the parameter identification.

## 6. Conclusions

This paper proposes a new method to detect the solenoid valve deterioration status for in-use electronic diesel fuel injection control systems without disassembling the pump. According to experiments the proposed diagnostic algorithm, which comprises a feedback controller, a parameter identifier, a LVDT sensor and a neural network classifier, performs with acceptable accuracy. The idea is to design a diagnostic device to identify the usability of the solenoid valve in the EDC system for service purposes. Furthermore, because of the non-destructivity, the proposed method can also be used for on-board monitoring applications in agricultural vehicles.

It should be noted that a neural network classifier is used for diagnosing the valve deterioration status only for demonstration purposes in the present study. Since the experimentally obtained decision boundary in our case was only a straight line, other methods such as the simple principal component analysis (PCA) technique can also be used in determining the decision boundary. Furthermore, the placement of decision boundary remarkably affects the trade-off between component replacement intervals and acceptable levels of exhaust emissions. A statistical validation with enough experimental data is required to determine the decision boundary. If possible, a larger data set should be used for the training of the artificial neural network to result in an optimal decision boundary.

## Figures and Tables

**Table 1.** Parameter changes of the solenoid valves.

	**Solenoid V_2_**	**Solenoid V_3_**
Δ*â_f_*/*â_f_*	52.45%	100.74%
Δ*a*_0_/*a*_0_	−2.5%	7.36%
Δ*a*_1_/*a*_1_	102.5%	316.9%
Δ*a*_2_/*a*_2_	−7.46%	−13.3%
